# Cyclo-oxygenase-2 (Cox-2) expression and resistance to platinum versus platinum/paclitaxel containing chemotherapy in advanced ovarian cancer

**DOI:** 10.1186/1471-2407-6-182

**Published:** 2006-07-11

**Authors:** Gabriella Ferrandina, Franco O Ranelletti, Enrica Martinelli, Amelia Paglia, Gian Franco Zannoni, Giovanni Scambia

**Affiliations:** 1 Gynecologic Oncology Unit, Catholic University, L.go Gemelli 8, 00168, Rome, Italy; 2 Institute of Histology, Catholic University, L.go A. Gemelli 8, 00168, Rome, Italy; 3 Institute of Pathology, Catholic University, L.go A. Gemelli 8, 00168, Rome, Italy; 4 Department of Oncology, Catholic University, Contrada Tappino, Campobasso, Italy

## Abstract

**Background:**

Cyclo-oxygenase-2 (COX-2), the key enzyme in the conversion of arachidonic acid to prostaglandins, is involved in critical steps of tumor onset and progression, and is a strong predictor of chemotherapy resistance and poor outcome in advanced ovarian cancer. To our knowledge, no data has been reported until now about the association between COX-2 status and response to different chemotherapy regimens.

**Methods:**

A retrospective study was performed to investigate the association of COX-2 with outcome and response to platinum versus platinum/paclitaxel in 68 primary ovarian cancer. COX-2 immunoreaction was performed on paraffin-embedded sections by using rabbit polyclonal antiserum against COX-2.

**Results:**

In the overall series, COX-2 positivity was found in a statistically significant higher percentage of not responding cases than in patients responding to chemotherapy (n = 15/21; 71.4% versus n = 17/47; 36.1%; p value = 0.0072). A higher percentage of COX-2 positivity was found in patients unresponsive (n = 11/13; 84.6%) versus patients responsive to platinum-based chemotherapy (n = 9/26; 34.6%). In cases administered platinum/paclitaxel, COX-2 positivity was found in 4 out of 8 (50%) of un responsive versus 8 out of 21 (38.1%) of responsive cases. Logistic regression analysis of parameters likely to affect response to treatment resulted in a p value = 0.17 for the interaction COX-2/type of treatment.

**Conclusion:**

Although these findings need to be confirmed in a larger series, our study suggests a possible indication that there is a difference in the influence of COX-2 on response depending on treatment regimen.

## Background

Ovarian cancer represents the fifth leading cause of death for cancer in women [1]. More than 70% of cases present with advanced stage of disease at diagnosis and despite advances in cytoreductive surgery and establishment of carboplatin/paclitaxel combination as the standard chemotherapy regimen, intrinsic or acquired tumor chemoresistance remains the major determinant of chemotherapy failure and unfavourable clinical outcome [1,2].

Several molecular alterations have been proposed to support tumor resistance to cytotoxic drugs, such as the expression of MDR phenotype, mutation of p53, bcl2 overexpression [3-5], but only very recently it has been recognized that specific molecular or biological profiles might characterize tumor sensitivity to classes of agents with different mechanisms of action. For instance, mutation of p53 has been demonstrated to be strictly associated with platinum resistance in *in vitro*, and preclinical models as well as in humans [4,6], while it is likely to sustain a higher chance of susceptibility to paclitaxel containing regimens [7,8]. Recently, expression of survivin, a member of the inhibitors of apoptosis protein [9], has been shown to be unrelated to response to cisplatin-based treatment, while its overexpression correlates with resistance to paclitaxel/platinum containing regimen in advanced ovarian carcinoma [10].

We showed that cyclooxygenase-2 (COX-2), the key enzyme in the conversion of arachidonic acid to prostaglandins, involved in critical steps of tumor onset and progression [10], is a strong predictor of chemotherapy resistance and poor outcome in advanced ovarian cancer, as well as cervical cancer patients [12-14]. These findings, also confirmed in other solid tumors, have also provided the rationale for the combination of selective COX-2 inhibitors with cytotoxic agents [15,16].

To our knowledge, no data has been reported until now about the association between COX-2 status and response to different chemotherapy regimens.

The aim of the study was to investigate the association of COX-2 with outcome and response to platinum versus platinum/paclitaxel containing regimens as first line treatment in a single institutional series of primary untreated advanced ovarian cancer patients.

## Methods

### Patient data

A retrospective analysis of 68 ovarian cancer patients admitted to the Gynecologic Oncology Unit, Catholic University of Rome was planned. The study was approved by our Ethical Committee. Staging was performed according to FIGO classification. In order to make the analysis of the association with response to chemotherapy and outcome the most reliable, a very homogenous series of stage IIIC-IV ovarian cancer patients with measurable disease after primary surgery were included in the study.

Within 2–3 weeks after surgery, all patients underwent 4 to 6 cycles of cisplatin-based (75–100 mg/m^2 ^for each cycle) or platinum/paclitaxel containing (175 mg/m^2 ^for each cycle) chemotherapy. Response to chemotherapy was specifically assessed by gynecological examination, total body CT scan, analysis of CA125 levels. Response to treatment was recorded according to WHO criteria [17], as follows: complete response was defined as disappearance of all clinically detectable disease lesions for at least 4 weeks; partial response was defined as more than 50% reduction in the sum of the products of the two largest perpendicular diameters of bidimensionally measurable lesions for at least 4 weeks; stable disease was defined as regression not meeting the aforementioned criteria for objective response; all other cases were considered to have progressive disease.

For the analysis of the association between COX-2 status and response to treatment patients were categorized in responsive (clinical complete and partial response) versus not responsive cases (no change/progression)

### Immunohistochemistry

COX-2 immunoreaction was performed as previously described [12,13]. Briefly, four μm sections of representative formalin-fixed paraffin-embedded blocks from each case were deparaffinized in xylene, rehydrated, treated with 0.3% H_2_O_2 _in methanol for 10 min, and subjected to heat-induced epitope retrieval in microwave oven using the Dako ChemMate detection kit (DAKO, Glostrup, Denmark). Slides were then simultaneously processed for immunohistochemistry on the TechMate Horizon automated staining system (DAKO) using the Vectastain ABC peroxidase kit (Vector Laboratories, Burlingame, CA). Sections were incubated with normal rabbit serum for 15 min, then with rabbit polyclonal antiserum against COX-2 (Cayman, Ann Arbor, MI, USA) diluted 1:300, for 1 h. Negative controls were performed using non immunized rabbit serum or by omitting the primary antiserum.

The laboratory analysis of COX-2 and evaluation of immunohistochemical results were done without any prior knowledge of the clinical parameters by two different pathologists (FOR, GFZ) by means of light microscopy. Proportion of immunostained cells was scored at low magnification (5 × objective lens) by evaluating the entire tumor area. In case of disagreement (n = 6/68, 8.8%), the sections were submitted to a rejoint evaluation. When tumor area with positive immunostaining was in excess of 10 % of total tumor area, the case was scored as positive.

### Statistical analysis

Fisher's exact test or χ^2 ^test were used to analyze the distribution of COX-2 positive cases according to clinico-pathological features and response to treatment.

Progression free survival (PFS) was calculated from the date of diagnosis to the date of progression or date last seen. Medians and life tables were computed using the product-limit estimate by the Kaplan and Meier method [18] and the log-rank test was employed to assess the statistical significance [19]. Stepwise logistic regression was used to analyze the role of clinico-pathological parameters as predictors of response to treatment and included the interaction COX-2/type of treatment.

Statistical analysis was carried out using SOLO (BMDP Statistical Software, Los Angeles, CA).

## Results

Table 1 summarizes the clinico-pathological characteristics of cases in the whole series and according to the type of first line chemotherapy agents.

In the overall series median age was 57 years (range: 28–81). Fifty cases (73.5%) were stage IIIC and 18 (26.5%) were stage IV disease.

Cytoreduction to residual tumor 0.5–1 cm was achieved in 18 (26.5%) of cases, while cytoreduction to residual tumor >1 cm was accomplished in 23 (33.8%) of cases. In 27 (39.7%) cases only multiple biopsies (exploratory laparotomy) were performed.

Overall, COX-2 positivity was documented in 32/68 cases (47.0%). In COX-2 positive cases the percentage of positive staining per total tumor area ranged from 15% to 45% (median value 20%). Although the use of raw data provides more information, we decided to use the cut-off value in order to define different risk patient categories. There was no difference in the distribution of age, stage, residual tumor, and histopathological findings, in cases administered platinum versus platinum/paclitaxel containing chemotherapy. As far as response to chemotherapy is concerned, in the overall series 47 patients (69.1%) were considered to be responsive to treatment, while 21 (30.9%) patients were classified as no responders (Table 2).

In Table 2 the association between COX-2 status and response to chemotherapy in the overall series is also summarized: COX-2 positivity was found in a statistically significant higher percentage of not responding cases than in patients responding to chemotherapy (n = 15/21; 71.4% versus n = 17/47; 36.1%; p value = 0.0072).

Logistic regression analysis including variables likely to affect response to treatment showed that the extent of residual tumor at surgery maintained an independent role as predictor of resistance to treatment (Table 3). The p value of 0.17 of the interaction COX-2/type of treatment suggests that the role of COX-2 on differential response depending on different treatments needs to be clarified in a larger series of cases.

As shown in Table 4, in the group of patients administered platinum-based chemotherapy, COX-2 positivity was found in 11 out of 13 (84.6%) of not responding patients versus 9 out of 26 (34.6%) of responding patients. On the other hand, in cases administered platinum/paclitaxel containing chemotherapy, COX-2 positivity was found in 4 out o 8 (50.0%) of unresponsive versus 8 out of 21 (38.1%) of responsive cases.

### Survival analysis

**Figure 1:**
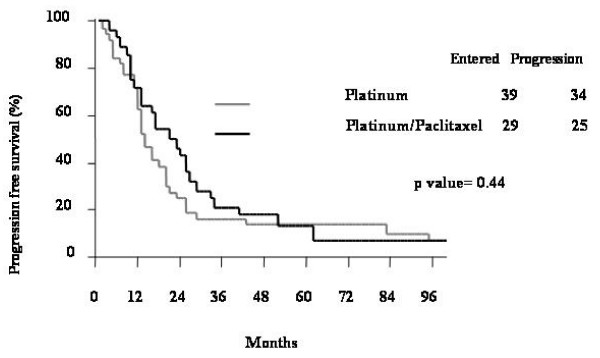
Progression free survival (PFS) curve in ovarian cancer patients administered platinum containing versus platinum/paclitaxel containing regimen.

Follow up data were available for 68 patients. The median follow up was 33 months (range 4–214). During the follow up period, progression of disease was observed in 59 (86.8%) cases. Figure 1 shows the progression free survival (PFS) curve in our population according to type of cytotoxic drugs in the first line treatment. There was no statistically significant difference of clinical outcome in patients administered platinum-based versus platinum/paclitaxel containing chemotherapy (p value = 0.44),

## Discussion

This is the first study investigating the association between COX2 status and susceptibility to chemotherapy according to type of cytotoxic agents used in first line treatment of advanced ovarian cancer with measurable disease after first surgery.

Our findings suggest that there could be a difference in the influence of COX-2 expression depending on type of chemotherapeutic regimen (a difference greater in platinum-based treated cases), although a larger sample series is needed before a definitive conclusion can be drawn.

Our data seems to confirm the association between high COX-expression and poor chance of response to chemotherapy reported in ovarian and cervical tumors [13,14,20]. This finding remains to be clarified at biochemical level, although it is conceivable that the involvement of COX-2 in the crucial biochemical pathways of tumor cell biology might play a major role: indeed, COX-2 has been reported to induce the antiapoptotic bcl-2 protein [21], and to be associated with neoangiogenesis in tumor bearing mice [22]. Since both inhibition of apoptosis and promotion of neoangiogenesis are strictly associated with chemotherapy resistance, it is conceivable that COX-2 expression could play a role as indicator of platinum resistance in ovarian cancer. Moreover, biochemical links between COX-2 and peptidic growth factor system alterations, which are renowned to be associated with resistance to several cytotoxic agents, have been documented [23,24]. Finally, very recent evidences demonstrated the relevant role of COX-2 in the inhibition of DNA damage induction of apoptosis mediated by p53 [25,26]; therefore, tumors overexpressing COX2 are likely to exhibit a reduction of wild-type p53 mediated apoptosis induced by DNA damaging chemotherapeutics, such as platinum compounds, alkylants and anthracyclines, often used in combination. Indeed, a direct association between high COX-2 content and overexpression/mutation of p53 has been reported in ovarian tumors, although conflicting data has been also provided [27-29].

Although the relatively small sample series has to be taken into account as a limit to the power of the analysis, it is conceivable that the addition of taxanes could hinder the COX-2 involvement in drug resistance, by acting on different molecular targets such as microtubule function or bcl-2 [30,31].

It remains to be clarified why there was no significant difference in terms of response rate and clinical outcome in patients administered platinum versus platinum/paclitaxel containing regimens: even if the impact of the relatively small size of our sample series cannot be ruled out, an alternative intriguing working hypothesis is represented by the interference of taxanes with COX-2 promoted pathways. Indeed, it has been reported that the two clinically available taxanes, namely paclitaxel and docetaxel increase COX-2 expression by inducing mRNA transcription and stabilization [32-35]. Moreover, the addition of the specific COX-2 inhibitor celecoxib has been shown to abrogate the marked increase of PGE_2 _levels observed in non small cell lung cancer patients after carboplatin/paclitaxel administration [36]. Whether the potentiation of COX-2 promoted activities by taxanes could influence the overall sensitivity of ovarian cancer cells to the combination platinum/paclitaxel, by conceivably reducing the favourable impact of paclitaxel addition, remains to be verified. In this context, it is noteworthy that the vast majority of the studies exploring the association of taxanes with COX-2 inhibitors were able to document a synergistic antitumor activity *in vitro *and in vivo [33-35].

Finally, although the significance of our findings is not certain given that the standard first-line treatment for ovarian cancer is a combination of platinum with a taxane, we think that our data could provide, if confirmed in a wider series, the rationale to ask for the immunohistochemical assessment of COX-2 status as predictor of response to treatment, only in cases triaged to platinum-based therapy (including platinum/anthracycline or platinum/alkylants combinations, which are still a valid option in selected patients).

## Conclusion

Although these findings need to be confirmed in a larger series, our study suggests a possible indication that there is a difference in the influence of COX-2 on response depending on treatment regimen. The analysis of COX-2 expression in ovarian cancer tissues before and after carboplatin/paclitaxel treatment in patients submitted to exploratory laparotomy and interval debulking surgery after successful neoadjuvant treatment, would be helpful in order to assess whether this regimen can modulate COX-2 expression *in vivo*, as very recently reported by Altorki et al. [37].

## Competing interests

The author(s) declare that they have no competing interests.

## Authors' contributions

GF conceived the study, was the coordinator of the study, and drafted the manuscript

FOR participated in the design of the study

EM carried out the immunohistochemical analysis

AP was responsible for clinical surveillance

GFZ was responsible for paraffin embedded sections and for the evaluation and scoring of the immunohistochemistry

GS participated in the coordination of the study, and in the conception and drafting of the study.

All authors read and approved the final manuscript.

## Pre-publication history

The pre-publication history for this paper can be accessed here:



## Figures and Tables

**Table 1:** Clinico-pathological characteristics of the patients in the whole series, and according to type of chemotherapy

**Characteristics**	**No. of patients**	**Platinum-containing chemotherapy No. (%)**	**Platinum/paclitaxel containing chemotherapy No. (%)**
All cases	68	39	29
Age (yrs)			
<60	43	22 (51.2)	21 (48.8)
≥60	25	17 (68.0)	8 (32.0)
FIGO Stage			
IIIC	50	31 (62.0)	19 (38.0)
IV	18	8 (44.4)	10 (55.6)
Cytoreduction			
RT <1 cm	18	12 (66.7)	6 (33.3)
RT ≥1 cm	50	27 (54.0)	7 (14.0)
Ascites			
No	16	8 (50.0)	8 (50.0)
Yes	52	29 (55.8)	23 (44.2)
Histotype			
Serous	54	32 (59.2)	22 (40.7)
Endometrioid	8	5 (62.5)	3 (37.5)
Undifferentiated	6	2 (33.3)	4 (66.7)
Other	3	2 (66.7)	1 (33.3)
Grade			
G1-2	10	8 (80.0)	2 (20.0)
G3	53	30 (56.6)	23 (43.4)
n.a.	5		
COX-2			
Negative	36	19 (52.8)	17 (47.2)
Positive	32	20 (62.5)	12 (37.5)

**Table 2:** Distribution of COX-2 positive cases according to response to chemotherapy

	**Overall series**		**P value**
Response to chemotherapy	No.	COX-2 positive No.(%)	

Responsive	47	17 (36.1)	
Not responsive	21	15 (71.4)	0.0072

*calculated by Fisher's exact test

**Table 3:** Logistic regression analysis of clinico-pathological parameters, and COX-2 status as predictors of response to treatment in ovarian cancer patients

Variable*	**χ**^**2 **^**estimate**	**p value**
Stage	0.11	0.7
Residual tumor	4.52	0.03
COX-2 status	2.04	0.09
Type of chemotherapy	2.10	0.11
Interaction	1.7	0.17

Response was subgrouped as follows: complete/partial vs no change of disease/progression.

*Variables were subgrouped as follows: stage III vs IV; residual tumor <2 cm vs ≥2 cm; negative vs positive COX-2 status; interaction COX-2 status/type of treatment

**Table 4:** COX-2 positive cases according to response to chemotherapy in the two treatment groups

	**Platinum-containing chemotherapy**		P value*	**Platinum/paclitaxel containing chemotherapy**		P value*
Response to CT	**No.**	**COX-2 positive No. (%)**		**No.**	**COX-2 positive No. (%)**	

Responsive	26	9 (34.6)		21	8 (38.1)	
Not responsive	13	11 (84.6)	0.0057	8	4 (50.0)	0.43

*calculated by Fisher's exact test
